# The Important Role and Implications of Citrate in the Composition, Structure, and Function of Oral/Periodontal/Craniofacial Tissues

**DOI:** 10.18689/mjdl-1000120

**Published:** 2018-08-17

**Authors:** LC Costello, RB Franklin, MA Reynolds

**Affiliations:** 1 Department of Oncology and Diagnostic Sciences, School of Dentistry, University of Maryland; and the University of Maryland Greenebaum Cancer Center, Baltimore, Md. 21201; 2 Office of the Dean of the School of Dentistry, University of Maryland, Baltimore, Md. 21201

**Keywords:** bone, dentin, cementum, enamel, osteogenesis, dentinogenesis, cementumogenesis, citrate in oral and periodontal tissues

## Abstract

High citrate concentration is a major component in the structure of craniofacial bone, teeth and periodontal tissues of humans and other osteovertebrates. It is now established that citrate incorporation into the apatite/collagen complex of bone is essential for the manifestation of the important biomechanical properties of bone; such as stability, strength, and resistance to fracture. The osteoblasts are specialized citrate-producing cells that provide the citrate incorporated in bone during osteogenic stem cell differentiation for production of new bone; “citration” that occurs in concert with mineralization. Dentin and cementum contain high citrate levels; as contrasted with low citrate in enamel. There exists no information regarding the status and source of incorporated citrate in dentin or in cementum. These are important issues relating to oral, periodontal, craniofacial structures. For example, repair of defects should include new tissue that exhibits the composition, structure, and biomechanical properties of the “normal” tissue; which cannot be achieved in the absence of citrate incorporation in the new tissues. Unfortunately, the presence and role of citrate in these tissues have been largely ignored and unrecognized over the past about 40 years by the dental and medical community. The intent of this review is to re-establish the interest and research regarding the important citrate relationships and issues; with focus on related interests in dentistry.

## Introduction

The American Academy of Orthopaedic Surgeons 2016 report [1] states; “*An estimated 126.6 million Americans (one in two adults) are affected by a musculoskeletal condition -- comparable to the total percentage of Americans living with a chronic lung or heart condition -- costing an estimated $213 billion in annual treatment, care and lost wages. As a nation, we need to establish greater funding for musculoskeletal research, improve our understanding and strategies for prevention and treatment of these injuries and conditions, and ensure that more adults and children receive appropriate treatment sooner, and on an ongoing basis, to ensure quality of life and productivity”*. Defects in the dental/periodontal/craniofacial complex present such an issue. Coinciding with that report, Elangovan and Allareddy in 2016 [2] reported that “*the dental community is noticing a surge in the number of complications associated with the use of dental implants. The complications that lead to implant failure can be…resulting either in progressive bone loss around implants or fracture of implant components*.”

In dealing with such issues the dental community must recognize the important role and implications of citrate relating to the composition, structure, and function of the oral/periodontal/ craniofacial tissues. Although the presence of high citrate levels in bone and in teeth has been known since 1941–1943 [3, 4], it has been ignored by the medical and dental community for the past ~40 years. Notably, citrate in bone and teeth is not mentioned in most contemporary textbooks and reviews; and it is not presented in lectures to medical and dental students, or to postgraduate students. Therefore, most descriptions and understanding of the composition and structure of normal bone and teeth are inaccurate; which leads to misrepresentations and questionable conclusions in issues relating to the development and maintenance of bone and teeth and their pathophysiological/clinical implications. This should no longer exist!

To address this, recent reviews [5–7] of citrate in bone were directed at the “medical community at-large”. Since issues of citrate in dental tissues were not included, this presentation is directed at the dental community. However, it will not include extensive reiteration of background information described in the earlier reviews. It will be advisable for the readers to avail themselves of those recent reviews.

## Status of citrate in the composition and structure of bone

That normal bone and teeth in humans and in all osteovertebrates contains high concentrations of citrate (Table 1) is well established [5]. However, its status in relation to other components of bone and in relation to the structure of bone had remained unsettled, until recent reported studies that have begun to establish the relationship of citrate in bone.

### Citrate in regard to the chemical composition of bone

The typical concentration of citrate in bone ranges from ~20–100 μmols/gram (micromoles/gram tissue weight); as compared to most other tissues in which the citrate concentration is ~0.2–0.4 μmols/gram [3, 4]. When adjusted for tissue water content (Table 1), bone contains ~25–75 fold higher citrate than most other tissues. Citrate comprises up to ~2 wt% of the total bone composition and ~5.5 wt% of the organic component of bone; and provides more carboxylate for calcium binding in bone than all of the non-collagen proteins combined. About 90% of the total citrate in the body resides in the bones. Thus, it is evident that citrate is a major component of the chemical composition of bone.

### Status of citrate in the structure of bone

Until recently, the status of citrate in the chemical and physical structure of bone had been speculative and disputed. In a recent report, NMR/x-ray diffraction analyses led Hu et al [8] to propose that citrate in bone is strongly bound to the apatite nanocrystal/collagen complex. In contrast, Davies et al. [9] proposed that citrate bridges the layers of mineral platelets that comprise the hydroxyapatite (mineral) component of bone.

**Figure1.**
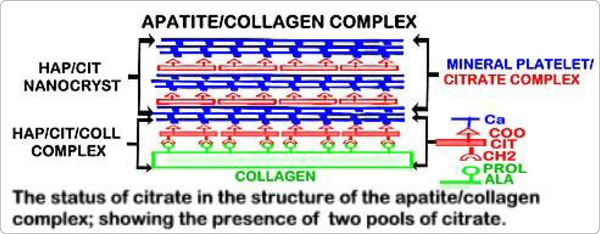
The incorporation of two pools of citrate in the structure of the mineralized hydroxyapatite/collagen structure of bone

Studies with human bone preparations [10] identified for the first time that the total concentration of bone citrate exists in two major pools (Table 2). In cortical bone, one pool of citrate is incorporated in the mineral component of bone and comprises ~66% of the total citrate. A second pool of citrate is strongly bound to the type I collagen complex and comprises ~33% of the total citrate. Cancellous bone contains a similar total citrate concentration that exists in the two major pools of citrate, except that the distribution is ~50% of the total citrate in each pool. Based on these relationships, the represented structure of bone as the apatite/citrate/ collagen structure of bone is shown in figure 1.

It is most important to recognize that contemporary descriptions and representations of the composition and structure of bone have excluded the incorporation of citrate as a major component. That is an untenable misrepresentation that should no longer continue.

### Importance of citrate in the structure of bone

The evolution of incorporation of citrate into the structure of bone and teeth of all osteovertebrates dictates that it must provide essential functional and/or structural properties of the bone. Normal bone exhibits important biomechanical properties including its stability, strength, and resistance to fracture. A major factor that is responsible for these properties is the structure, size and orientation of the apatite nanocrystal/ collagen complex. Both Hu et al. [8] and Davies et al. [9] described that the optimal thickness of the nanocrystal/collagen structure is established by the appropriate incorporation of citrate. In the absence of citrate incorporation, mineralization continually increases and the biomechanical properties of bone are compromised. This is supported by the report of Caudarella et al. [11] that the “***citrate level in bone is the most prevalent biochemical parameter that is best related with vertebral fractures (p=0.001) in osteopenic (preosteoporotic) women*”**. Similarly, VitD-deficient rachitic bone is characterized by increased fracture; and VitD treatment restores citrate production in bone (described below). These implications of citrate in bone disorders characterized by loss of bone strength and increased fractures have been largely ignored.

### Osteoblast citrate production is the source of citrate that is incorporated into bone

**Figure 2.**
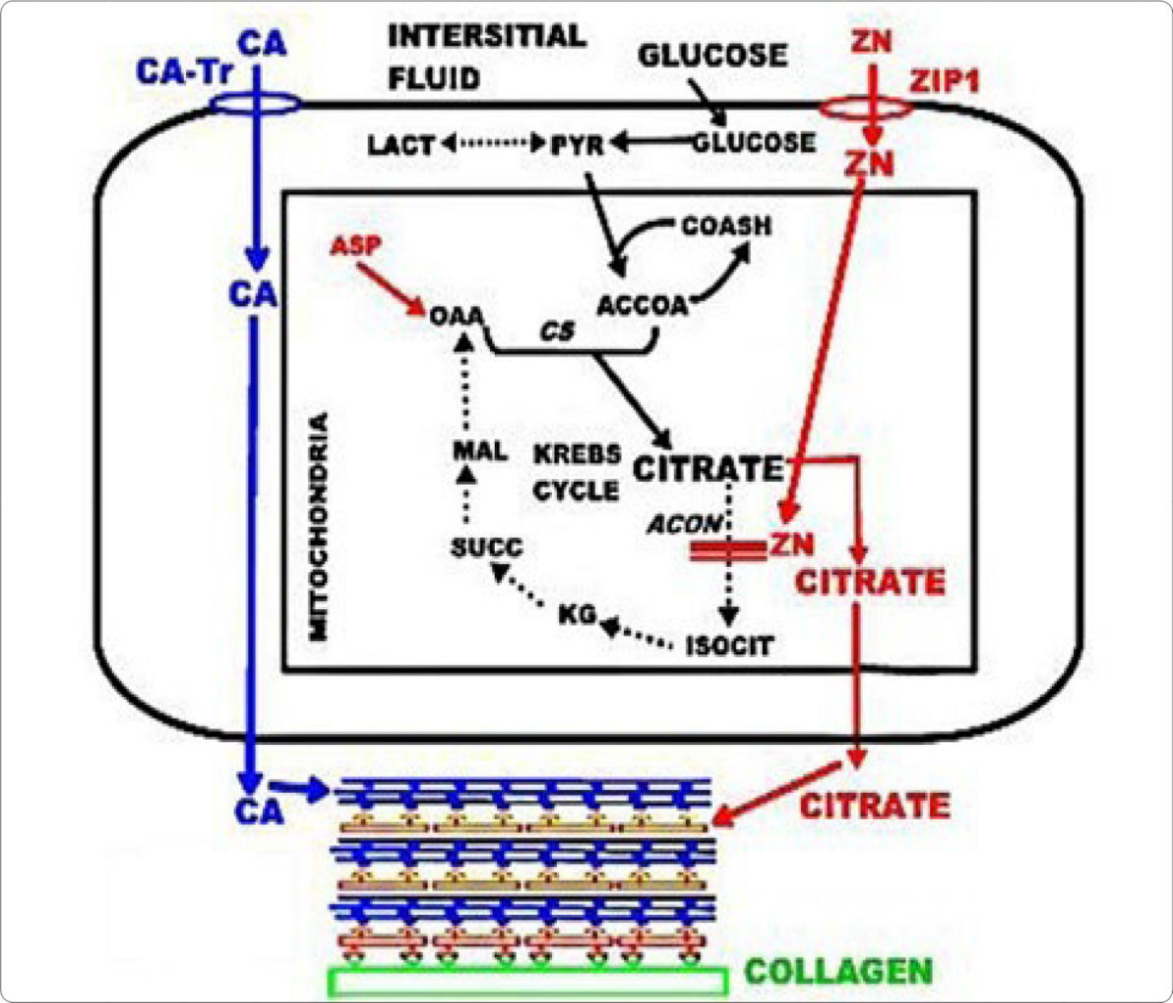
The unique metabolic pathway of specialized citrate-producing osteoblasts. Blue shows Ca-transporter (CA-Tr). Red shows ZIP1 zinc uptake of zinc, which inhibits m-aconitase activity so that citrate oxidation via the Krebs cycle is aborted; and the citrate is incorporated into bone

For 70 years, the source of citrate for incorporation into bone had remained unknown. Recent studies [5, 12, 13] identified the osteoblasts as specialized citrate-producing cells that provide the citrate for incorporation into bone. In typical mammalian cell metabolism, the citrate in cells is produced in the mitochondria for entry into the Krebs cycle and oxidized as the major source for ATP production. The secretion of citrate out of the cell occurs only in specialized “citrate producing” cells; which requires significant alteration in the citrate metabolism of typical mammalian cells. This is now referred to as “net citrate production”. Costello et al. [14] identified the metabolic pathway in citrate-producing prostate epithelial cells; and it also occurs in osteoblasts (figure 2). It is important to note that the osteoblast production of citrate for “citration”, and Ca transport from plasma for mineralization, are separate events during bone formation. Consequently, a new understanding now exists, which differs from the prevailing view that citrate is transported along with calcium (CaCit) from plasma into bone during bone formation.

### Citrate incorporation (“citration”) in bone is an essential event in osteogenesis

**Figure 3.**
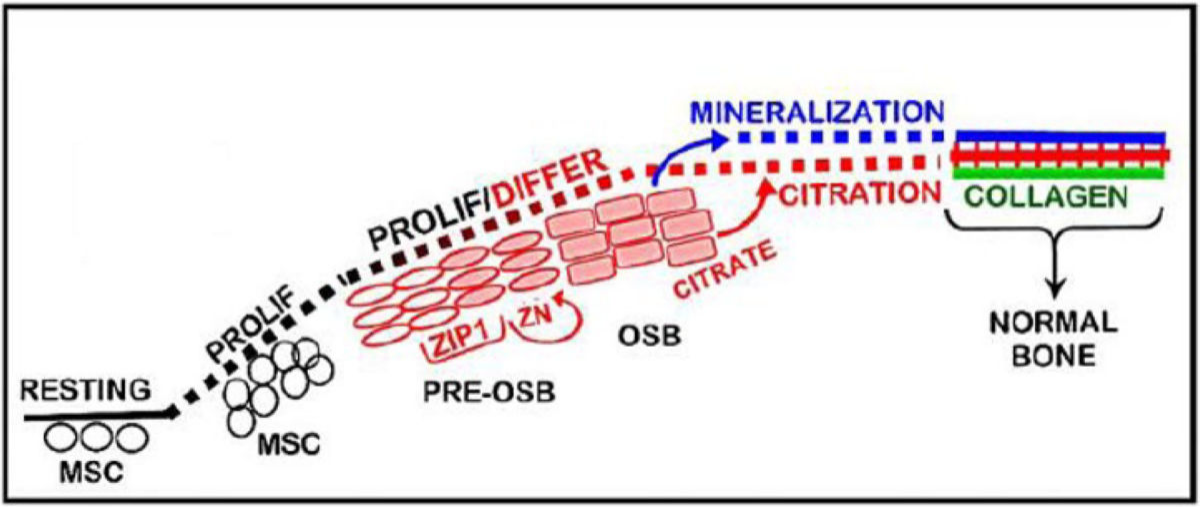
Osteogenic differentiation of mesenchyme stem cells to specialized citrate-producing osteoblasts during the process of new bone formation. Citrate incorporation (“citration”) occurs in concert with mineralization

These citrate relationships dictate the necessity for a new and more correct understanding of the process of osteogenesis; which, until now, has excluded the requirement for citrate incorporation in the development of new bone. Notably, human and murine mesenchyme stem cells in osteogenic medium supplemented with physiological levels of zinc and aspartate, differentiate into citrateproducing osteoblasts for incorporation into bone (i.e. “citration”) along with the mineralization of the bone nodules [12, 13]. Up regulation of ZIP1 zinc transporter (figure 3) is required. The increased transport and increased cellular zinc results in inhibition of m-aconitase [14] and decreased utilization of citrate by the Krebs cycle. Thus, citrate is available for bone “citration”; and presents a new and more correct understanding of osteogenesis and the structure of normal bone that exhibits the important biomechanical properties. “***Mineralization without citration does not produce bone that exhibits the structure and biomechanical properties of normal bone***”.

## Status of citrate in the composition and structure of teeth

The above citrate relationships in bone must be considered in relation to teeth and periodontal tissues.

### Citrate concentrations in teeth, periodontal tissue, craniofacial bone

In 1943, Free [4] reported that animal and human teeth and periodontal tissue contained high citrate levels; due to the high citrate concentrations in dentin and cementum. In contrast, enamel contains low citrate concentration. Table 3 provides representative citrate values derived from several reports [4, 15–21].

Notably, enamel is the hardest and most mineralized structure in the human body. It consists of ~95 wt% mineral in the form of hydroxyapatite; and contains no collagen and low citrate. Thus the apatite /citrate/collagen complex does not exist in enamel.

### Status of citrate in the structure of dentin

The distribution of citrate in dentin has not been established. The early studies of Hartles et al. [18–20] led to their conclusion that the citrate in dentin was mainly strongly bound to a non-collagen peptide of unknown origin. However, they also reported that the same relationship existed in their analysis of the citrate status in ox bone. The available methodologies at that time did not permit the more precise and specific extraction capabilities and analyses that currently exist. The more recent study [10] clearly established that complete demineralization of bone under mild conditions results in the isolation of in-tact tightly bound “Ca/citrate/ type I collagen complex” (figure 1).

Hu et al. [8] described that citric acid-CH2 binds to collagen non-polar hydrophobic amino acids (e.g. proline, alanine); and the citric acid-COOH binds to Ca; which together result in the Ca/citrate/collagen complex as represented in figure 1. The mineral and organic composition of dentin exhibit similarities to bone; especially that type I collagen constitutes ~90% of its organic component. It seems likely that a citrate component of dentin would be tightly bound in a Ca/citrate/collagen complex similar to normal bone. The studies of Davies et al. [9] provide evidence that citrate anions bridge between the layers of apatitic platelets that comprise the mineral component of bone (figure 1). Costello et al. [10] established that citrate exists in both components of the apatite nanocrystal/collagen structure of bone. Hu et al. [8] and Davies et al. [9] agree that the citrate incorporated into the structure of bone is essential to limit the crystallinity of the mineral component, since greater crystallinity and larger mineral crystals are associated with greater fragility and increased fracture of bone. Since these properties are also important for dentin, it is likely that citrate incorporation in dentin is similar to bone. It is now most important to investigate and establish the status of citrate in the structure of dentin as we had achieved for bone.

### Status of citrate in the structure of cementum

A search of Google Scholar and PubMed did not identify any reports of the status and distribution of citrate in cementum. Since the mineral and collagen content is similar to bone, we speculate that the distribution of the high citrate concentration is distributed in the mineral/collagen complex as we discussed for dentin and exists in bone. This would manifest the properties of stability, strength, flexibility, and resistance to fracture; which are important for the role of cementum in the periodontal complex. Studies to establish the distribution and status of citrate in the structure of cementum are essential.

## Source of citrate incorporated into dentin and cementum

The osteoblasts are specialized citrate-producing cells that provide the citrate that, in concert with mineralization (figures 2, 3) achieves the required timing and amount of citrate incorporated into the mineral/collagen complex; thereby providing the optimal thickness for the biomechanical properties. It is reasonable to expect that the odontoblasts and the cementoblasts are also specialized “citrate-producing cells” that produce citrate that is incorporated into the mineral/collagen complex of dentin and cementum, respectively. Unfortunately, a search of the literature did not identify any reports regarding the source of citrate in dentin or in cementum. It is important to recognize that studies of citrate production and incorporation into dentin and cementum during the development of odontoblasts and cementoblasts must include conditions that are required for the cellular production of citrate; such as physiological concentrations of zinc (~5–10μM) and aspartate (~30–50μM). Without such conditions, the absence of citrate production could result in a misleading conclusion that the cells do not produce citrate.

### Odontoblasts and odontogenesis; the source of citrate in dentin?

There are important differences between osteogenesis and dentinogenesis that impact the citrate relationships. For example, bone turnover (formation←→ resorption) and remodeling occur throughout the lifetime; and requires that osteogenesis along with its production of new populations of citrate-producing osteoblasts is a continuing process. In contrast, the induction of odontogenesis leading to the production of the odontoblasts occurs only during tooth development; and the odontoblasts survive throughout the lives of healthy teeth. If damaging conditions destroy the odontoblasts, the differentiation of dental pulp stem cells into odontoblast-like cells is induced, and reparative dentin is produced. The composition of the new dentin exhibits differences from the original dentin [22, 23]. Consequently, the status and implications of citrate needs to be considered and determined for odontogenesis, for odontoblast production of dentin during the development of the teeth; and also for the “odontoblast-like” cell production of reparative dentin. Also, the histological organization and process of odonotogenesis→dentinogenesis are more complex than osteogenesis [24, 25].

These relationships raise citrate issues that are not involved in osteogenesis and osteoblast activity. Do the odontoblasts provide the citrate that is incorporated into dentin? If so, are the odontoblasts specialized citrate-producing cells? If so, does dentinogenesis involve the transformation of the dental pulp stem cells into citrate-producing odontoblasts for citration in concert with mineralization? Do these events also occur during the process of post-development reparative dentin production? These and related issues of citrate incorporation in dentin need to be recognized, investigated, and elucidated.

### Cementogenesis and cementoblasts: The source of citrate in cementum?

Following the identification of citrate in cementum from ~1943–1960, no reported studies exist of citrate relationships in cementum; so that the source of citrate in cementum has remained unknown. There exist important unsettled issues regarding the identification of cementoblasts and the process of cementogenesis leading to the development of the cementoblasts. This is further complicated by the existence of different forms of cementum. Prevailing evidence exists that periodontal ligament stem cells (PDLSC) include the capability of differentiating into cementoblasts and the production of cementum [26–29]. However the specific mesenchyme cell line has not been established.

Such unsettled issues make it difficult to determine the existence of a cementogensis process in which stem cell differentiation leads to the development of cementoblasts and the production of citrate that is incorporated into the composition and structure of cementum. Indeed, if cementum includes citrate incorporated into the mineral/collagen structure, this would be an important “biomarker” for identification of progenitor cells that differentiate into cementoblasts. However, such studies must include conditions that provide the physiological conditions (described above), which are required for production of high citrate levels (figures 2 and 3).

## The pathological implications of citrate dysregulation in oral/dental/craniofacial disorders

The implications of citrate in oral/periodontal/craniofacial defects must be addressed. Its importance is recognized in the 2016 report [2] that “*the dental community is noticing a surge in the number of complications associated with the use of dental implants*. *The complications that lead to implant failure can be… resulting either in progressive bone loss around implants or fracture of implant components*”. Highly relevant to the public health issue is that *“An estimated 126.6 million Americans (one in two adults) are affected by a musculoskeletal condition -- comparable to the total percentage of Americans living with a chronic lung or heart condition... As a nation, we need to establish greater funding for musculoskeletal research, improve our understanding and strategies for prevention and treatment of these injuries and conditions, and ensure that more adults and children receive appropriate treatment sooner, and on an ongoing basis, to ensure quality of life and productivity* [1].

Decreased citrate is a major factor in bone disorders that exhibit loss of strength and increased fracture; and agents that restore the citrate content in bone are effective in treating these conditions [7]. The biomechanical properties are essential to maintain the normal structure and function of the teeth and orofacial components and resist the potential trauma and damage that are imposed by the forces of mastication. So, the citrate relationships expectedly also apply to dentin and cementum. Therefore the periodontal and dental pulp components are maintained and protected by the citrate/ mineral/collagen structure in dentin and in cementum; and in the repair process in response to damage of these structures. Unfortunately, most of the contemporary clinical community have not recognized and considered these citrate relationships.

## Vitamin D-deficient rickets and rachitic teeth

In the important 2006 report “Resurrection of vitamin D deficiency and rickets”, Holick [30] states that “*Vitamin D deficiency has again become an epidemic in children, and rickets has become a global health issue*”. Foster et al in 2014 [31] provide an excellent comprehensive review and description of the “Rachitic Tooth” and the role of VitD. However, the citrate implication in VitD-deficient rickets was not addressed.

Early studies had well established that VitD is an important hypercitricemic agent, and also increases citrate levels in bone and kidney [7]. The relationship of citrate in VitD-deficient rickets was also a focus of early studies. Rachitic bone is characterized by increased fragility and fractures. Rachitic children exhibit hypercitricemia and VitD treatment restores the normal plasma citrate concentration; and also restores the bone strength and resistance to fracture. Such a relationship is a likely factor in rachitic teeth, which would be due to incorporation of citrate in the mineral/collagen structure of dentin. VitD deficiency would also impact the periodontal bone to exhibit decreased citrate and exhibit change in bone strength and susceptibility to fracture; and perhaps cementum as well.

VitD treatment is also employed for the treatment of osteoporosis, in which decreased citrate is a major factor associated with increased fractures; but the VitD citrate relationship has not been recognized by contemporary clinicians and biomedical investigators yet. The identification of the mechanism involved in VitD promotion of citrate production in various tissues needs to be established.

## The implications of citrate to the repair and replacement of dental/periodontal/ craniofacial defects

Regenerative medicine for the repair and replacement of dental/periodontal/craniofacial defects offers the opportunity “*to achieve successful treatment outcomes from a functional, esthetic and phonetic point of view with high predictability and good long-term stability; and to have a low risk of complications during healing and during the follow-up period*” [32].

A fundamental requirement is that the composition, structure, and functional properties of the regenerated product (bone, dentin, cementum) should represent the status that exists in the normal product. Appropriate conditions must exist, which are necessary to promote the production and incorporation of citrate in the structural composition of these products; and to optimize the biomechanical properties. In the absence of such conditions, the product will exhibit adverse conditions such as loss of strength, increased brittleness and fracture, incompatibility with adjoining tissues.

## Summary and Conclusions

This review has focused on the important role and implication of citrate in normal and defective bone, dentin, and cementum as related to dental/periodontal/craniofacial tissues. The presentation included the description of important established relationships of the status of citrate; along with speculation and concepts of the implications of citrate in the normal development of these tissues; in disorders and defects; and in their treatment. Some of the description is speculative due to the absence of recognition, interest, and research regarding the existence and implications of citrate by the medical/dental community for the past ~40 years. Thus, progress has been deterred in correctly identifying the factors and conditions involved in the normal development and composition of these tissues; in identifying and assessing the factors involved in their disorders; and in developing efficacious treatment and remedy of the disorders and defects.

Hopefully, this review will bring attention and generate interest and research into the important role and implications of citrate, which is in the best interest of the dental/medical community and the health and welfare of the public.

## Figures and Tables

**Table 1.** Typical citrate levels in bone and other tissues.*

	mM Citrate
Bone/teeth**	5–30
Cartilage	0.5–2
Other tissues	0.2–0.4
Prostate tissue**	10–15
Blood plasma	0.12

* values are based on water content of the tissues.

** citrate-producing tissue

**Table 2.** Citrate, calcium, collagen status in human cortical bone.

	(% of total)		
BONE COMPONENT	CIT	CA	COLL
APATITE (MINERAL)	66	90	<1
COLLAGEN COMPLEX	33	<1	99
OTHER	1	10	<1
TOTAL	100	100	100

Other: collagen complex extract

**Table 3.** Relative citrate content of dental/oral/facial tissue (% dry wt)

Facial Bone	1.20
Teeth	0.70
Enamel	0.10
Dentin	0.90
Cementum	0.90
Cartilage	0.10
Muscle	0.04
